# Fungoid Pulp

**Published:** 1900-11

**Authors:** R. L. Gooding


### FUNGOID PULP.

BY R. L. GOODING, D.D.S.

On Thursday, January 19, Miss C., aged ten years, came to the clinic for treatment. On examining her mouth, I observed her teeth were very irregular and carious, and in the right lower first permanent molar there was a cavity of considerable size, the lingual and distal sides of which were broken down, and a clear case of polypus or hypertrophied pulp presented itself. The case was diagnosed as being one of that character, and I proceeded with my treatment. To apply the rubber dam was impossible, so resort had to be made to the napkin. I isolated the tooth as well as I could and applied trichloracetic acid crystals, burning off a great portion; then, with my engine-bur and excavators, removed as much as possible. At this period of the operation I observed my patient was getting faint, so I concluded by placing in the cavity cotton saturated with iodine, and then a temporary stopping.

On her return two days later the previous dressing was removed and arsenic fibre was applied, and Robinson's remedy, hermetically sealing the cavity. Her next visit was on the 23d, when I found that the arsenical application had had no effect, and the part was exceedingly painful and congested, so I applied acetate of morphia and oil of cloves, and had her return on the 27th. This time I made an application of a double dose of arsenic, using the crystals along with morphine acetate and oil of cloves. The next day I removed this application, and found that the death of the pulp was complete. All putrescent matter was removed, the canals thoroughly cleaned in the usual manner, and a dressing of Black's 1, 2, 3 placed in.

I had her return at different intervals, when I renewed the dressing, and on February 21, finding the conditions perfectly healthy, I filled the canals with oxychloride of zinc and the cavity with amalgam.

[Remarks.—There is a general feeling prevalent in the dental profession that it is a hopeless waste of time to treat fungoid or hypertrophied pulps. Until of recent years teeth thus affected were universally condemned to the forceps. Various methods have been devised to destroy this character of pulp, but only with limited success. Some years ago Dr. Maercklein, of Milwaukee, announced that they could be removed by the application of crystals of iodine. This was tried in the clinic of the Dental Department of the University of Pennsylvania, with some modifications, and found to be very satisfactory. The papers given were cases in that clinic. These were selected from a number as typical of the method at present used.—Editor.]

